# Zinc Status Index (ZSI) for Quantification of Zinc Physiological Status

**DOI:** 10.3390/nu13103399

**Published:** 2021-09-27

**Authors:** Jacquelyn Cheng, Haim Bar, Elad Tako

**Affiliations:** 1 Department of Food Science, Cornell University, Stocking Hall, Ithaca, NY 14853, USA; jyc53@cornell.edu; 2 Department of Statistics, University of Connecticut, Philip E. Austin Building, Storrs, CT 06269, USA; haim.bar@uconn.edu

**Keywords:** zinc biomarker, microbiome, LA:DGLA, ∆6-desaturase, zinc deficiency, zinc transporters

## Abstract

Zinc (Zn) deficiency is estimated to affect over one billion (17%) of the world’s population. Zn plays a key role in various cellular processes such as differentiation, apoptosis, and proliferation, and is used for vital biochemical and structural processes in the body. Widely used biomarkers of Zn status include plasma, whole blood, and urine Zn, which decrease in severe Zn deficiency; however, accurate assessment of Zn status, especially in mild to moderate deficiency, is difficult, as studies with these biomarkers are often contradictory and inconsistent. Thus, sensitive and specific biological markers of Zn physiological status are still needed. In this communication, we provide the Zn status index (ZSI) concept, which consists of a three-pillar formula: (1) the LA:DGLA ratio, (2) mRNA gene expression of Zn-related proteins, and (3) gut microbiome profiling to provide a clear assessment of Zn physiological status and degree of Zn deficiency with respect to assessing dietary Zn manipulation. Analysis of five selected studies found that with lower dietary Zn intake, erythrocyte LA:DGLA ratio increased, mRNA gene expression of Zn-related proteins in duodenal and liver tissues was altered, and gut microbiota populations differed, where the ZSI, a statistical model trained on data from these studies, was built to give an accurate estimation of Zn physiological status. However, the ZSI needs to be tested and refined further to determine its full potential.

## 1. Introduction

Over one billion people worldwide (17% of the global population) suffer from dietary zinc (Zn) deficiency [1]. Zn is vital for numerous physiological and metabolic processes and plays a key role in various cellular processes such as differentiation, apoptosis, and proliferation [2]. Zn is a required cofactor for the function of over 300 different enzymes in the human body and approximately 10% of all human proteins presumably bind Zn in vivo [3,4]. Consequently, Zn has been implicated in key functions in the nervous, reproductive, and immune systems, and plays a central role in growth and development, where Zn inadequacy has been associated with poor growth, depressed immune function, increased vulnerability to and severity of infection, adverse outcomes of pregnancy, and neurobehavioral abnormalities [2,5,6]. As deficiency of Zn has been linked to severe health consequences, it is a major cause of early childhood morbidity and mortality in developing nations [7].

In the past decades, there has been a significant increase in the understanding of Zn homeostasis; however, an accurate assessment tool for Zn status remains elusive. Currently, there is no universally accepted single measure to assess Zn status. Widely used biomarkers of Zn status include plasma, whole blood, and urine Zn, which decrease in severe Zn deficiency; however, accurate assessment of Zn status, especially in mild to moderate deficiency, is difficult, as findings from studies with these biomarkers are often contradictory and inconsistent [8]. The World Health Organization (WHO) has estimated that one-third of the global population is at risk for Zn deficiency based on the calculated proportion of individuals with intakes below country-level daily Zn requirements [9,10]. To recognize Zn deficiency in its early states, the WHO has indicated a need to develop additional robust indicators of Zn status and to further expand on already known clinical markers. Emerging biomarkers of Zn status that require further investigation include Zn-dependent proteins, Zn kinetics, taste acuity, oxidative stress, and DNA integrity [11].

In recent years, evidence has suggested current biomarkers, such as plasma (or serum) Zn, are not sensitive and specific enough to small changes in Zn nutrition due to the ubiquitous nature of Zn in human biological systems [11,12]. Previously, our group established the concept of the essential role of Zn for ∆6-desaturase activity, where we explored Zn status relative to erythrocyte ∆6-desaturation, the LA:DGLA (linoleic acid:dihomo-γ-linolenic acid) ratio. We evaluated and provided evidence that demonstrated the effectiveness of the LA:DGLA ratio as a sensitive biomarker for assessing Zn status, where a significant negative correlation was found between dietary Zn intake and the LA:DGLA ratio [13]. Mild Zn deficiency has been shown to alter Zn transporter (ZIP and ZnT transporters) gene expression and brush border membrane enzyme activity (∆6-desaturase) in vivo [14,15,16]. Further, the intestinal microbial environment is crucial for Zn metabolism and is in turn influenced by inferior Zn status. Previous work has demonstrated that lack of dietary Zn deleteriously affects the composition of the intestinal microbial populations through reductions in taxonomic richness and diversity, decreases in beneficial short-chain fatty acid (SCFA) production, and changes in the metagenomic potential of the microbiota [17,18]. Given that these perturbations may serve as possible effectors of Zn deficiency physiological status by limiting Zn solubility and precluding the host from optimal Zn availability, it is critical to consider these factors in relation to Zn physiological status [17,19].

Considering the complexity of Zn metabolism, establishing a panel of biochemical indices is necessary to reliably assess Zn status. We have further developed a Zn status index (ZSI), a three-pillar formula that consists of (1) the LA:DGLA ratio, (2) mRNA gene expression of Zn-related proteins, and (3) fecal microbiome profiling to provide a clear and accurate measurement of Zn physiological status. Our ZSI aims to improve the understanding of Zn nutrition, physiological status, and severity of potential deficiency, which will ultimately lead to effective dietary Zn interventions and medical outcomes [13,18,20]. In this manuscript, we will first review the literature that discusses the rationale behind the three pillars of the ZSI, and then discuss the development and usage of the ZSI.

## 2. Review of Literature on the Three Pillars of the ZSI: LA:DGLA Ratio, Zn-Related Gene Expression, and Gut Microbiome Modulation

Five selected studies (Reed et al., 2014; Reed et al., 2015; Knez et al., 2017; Reed et al., 2018; and Beasley et al., 2020) were analyzed for this communication [13,17,18,20,21]. These studies utilized diet-controlled experiments with differential Zn content and determined how differential dietary Zn could affect the combination of parameters used in the ZSI. Due to the paucity of data that examined the combination of the LA:DGLA ratio, Zn-related gene expression, and gut microbiome, all five studies discussed utilized the *Gallus gallus* in vivo model and were selected on the basis that the data were readily available (studies were conducted in the authors’ lab). The *Gallus gallus* model has previously been used to assess mineral bioavailability due to its sensitivity to dietary manipulation of minerals, such as Zn, and thus can serve as a model for dietary Zn bioavailability and absorption in humans [22,23,24,25,26]. There is also >85% homology between human and *Gallus gallus* in intestinal genes responsible for the expression of BBM (brush border membrane) proteins involved with mineral absorption, such as Zn Transporter 1 (ZnT1) [27]. Additionally, the *Gallus gallus* model harbors a complex and active gut microbiome, with significant resemblance at the phylum level between the gut microbiota of *Gallus gallus* and humans, with *Bacteroidetes*, *Firmicutes*, *Proteobacteria*, and *Actinobacteria* representing the dominant bacterial phyla in both [25,28,29].

### 2.1. Materials and Methods

#### 2.1.1. Animal Model, Study Design, and Experimental Diets

Cornish cross-fertile broiler chicken eggs were obtained from a commercial hatchery (Moyer’s chicks, Quakertown, PA, USA). The eggs were incubated under optimal conditions at the Cornell University Animal Science poultry farm incubator until hatching [30]. Hatchlings were randomly distributed into two treatment groups based on body weight and sex to ensure equal distribution between groups. Chickens were housed in cages (1 m^2^) and provided ad libitum access to food and H_2_O. Reed et al. (2014) and Reed et al. (2015) were conducted over the course of 4 weeks. Knez et al. (2017), Reed et al. (2018), and Beasley et al. (2020) were conducted over the course of 6 weeks. At the study conclusion, the animal subjects were euthanized by CO_2_ exposure, and the ceca, duodenum, and liver were quickly removed and stored in a −80 °C freezer until analysis, as was previously described [13]. All animal protocols were approved by Cornell University Institutional Animal Care and Use Committee (IACUC #2020-0077).

The NRC (Nation Research Council) recommendations and requirements for poultry were consulted to formulate diets that meet the nutrient requirements for the broiler [31]. In Reed et al. (2014) and Reed et al. (2015), the experimental diets (Zn-adequate control and Zn-deficient groups) differed only in terms of supplemental Zn (as Zn carbonate) [13]. In Knez et al. (2017) and Reed et al. (2018), the wheat-based diets (standard and Zn-biofortified wheat) differed only in levels of Zn. In Beasley et al. (2020), the wheat-based diets (standard and nicotianamine enhanced Zn- and Fe-biofortified wheat) differed only in levels of Zn, Fe, and nicotianamine. In the Knez et al. (2017), Reed et al. (2018), and Beasley et al. (2020) studies, Zn, Fe, phytate, calcium, fatty acid, and protein concentrations were measured in the standard and biofortified wheat-based diets as previously described [13]. Further details on the diet preparation and diet composition can be found in the respective studies [13,17,18,20,21].

#### 2.1.2. Blood Collection and Erythrocyte Fatty Acid Analysis

Blood was collected weekly from the wing vein using micro-hematocrit heparinized capillary tubes (Fisher Scientific, Waltham, MA, USA) following an 8 h overnight fast. The blood samples were stored on ice until transportation within 4 h to the Tako Laboratory, where whole blood was fractionated by centrifuging at ~2000× *g* for 10–15 min at room temperature and stored in a −80 °C freezer until analysis. Fatty acid profile was determined via gas chromatography mass spectrometry after fatty acid extraction from blood erythrocytes and derivatization to fatty acid methyl esters with boron trifluoride in methanol. The method for erythrocyte fatty acid analysis was previously described [13,32,33,34,35].

#### 2.1.3. Determination of Serum, Nail, Feather, and Liver Zn Content

Blood, nail, feather, and liver samples were collected on the final day of the experiment (~1–2 g). Serum, nail, and feather Zn concentrations were determined by an inductively coupled argon-plasma/atomic emission spectrophotometer (ICAP 61E Thermal Jarrell Ash Trace Analyzer, Jarrell Ash Co., Franklin, MA, USA) following wet ashing as previously described [13,21].

#### 2.1.4. Isolation of Total RNA

Total RNA was extracted from 30 mg of duodenal or liver tissue using a Qiagen RNeasy Mini Kit (Qiagen Inc., Germantown, MD, USA) according to the manufacturer’s protocol. Total RNA was eluted in 50 μL of RNase-free water. All steps were carried out under RNase-free conditions. RNA was quantified with a NanoDrop 2000 (ThermoFisher Scientific, Waltham, MA, USA) at A_260/280_. RNA was stored at −80 °C until use.

#### 2.1.5. Real-Time Polymerase Chain Reaction (RT-PCR)

Primer design was conducted as previously published [36]. The sequence and primer description are shown in Table 1. cDNA was generated using a C1000 Touch thermocycler (Bio-Rad, Hercules, CA, USA) and a Promega-Improm-II Reverse Transcriptase Kit (Catalog #A1250) 20 μL reverse transcriptase reaction. The reverse transcriptase reaction consisted of 1 μg total RNA template, 10 μM random hexamer primers, and 2 mM of oligo-dT primers. All reactions were performed under the following conditions: 94 °C for 5 min, 60 min at 42 °C, 70 °C for 15 min, and hold at 4 °C. The concentration of cDNA obtained was determined with a NanoDrop 2000 at *A_260/280_* with an extinction coefficient of 33 for single-stranded DNA. RT-PCR was performed as previously published [21,37].

#### 2.1.6. 16S rRNA Gene Amplification, Sequencing, and Analysis

16S rRNA gene amplification, sequencing, and analysis were performed as previously described [16,17,18,20,29]. Microbial genomic DNA was extracted from cecal samples using the PowerSoil DNA isolation kit, as described by the manufacturer (MoBio Laboratories Ltd., Carlsbad, CA, USA). Bacterial 16S rRNA gene sequences were PCR-amplified from each sample using the 515F-806R primers for the V4 hypervariable region of the 16S rRNA gene, including 12-base barcodes. 16S rRNA gene sequence analysis was performed as previously described [17].

#### 2.1.7. Statistical Analysis

Dissimilarities among experimental groups were tested by ANOVA using SAS software (SAS Institute Inc. Cary, NC, USA) with Tukey’s method for adjustment for multiple testing. A threshold of adjusted-*p* < 0.05 was considered statistically significant. Nonparametric factorial Kruskal–Wallis rank-sum tests were used to compare the relative abundance of distinct taxonomic units. Unweighted UniFrac was used to assess phylogenetic diversity. The Spearman’s rank correlation was employed to assess significant associations between bacterial groups and biomarkers of Zn status. Multivariate Association with Linear Models (MaAsLin) was used to identify potential correlations between operational taxonomic unit (OTU) abundance and host phenotype. Significant *p*-values (*p* < 0.05) associated with microbial clades and functions identified by Linear discriminant analysis Effect Size (LEfSe) were corrected for multiple comparisons using the Benjamini and Hochberg false discovery rate (FDR) method.

### 2.2. Results

The five original papers selected and included for the ZSI were performed in the United States and the experimental model used was the Cornish Cross broiler (*Gallus gallus*) [13,17,18,20,21].

All the studies were based on diets consisting of differing amounts of dietary Zn. The Zn dosage varied between studies, as summarized in Table 2. One study (Beasley et al., 2020) evaluated nicotianamine-enhanced Zn- and Fe-biofortified wheat effects in the context of a complete diet composed of 80% wheat on the LA:DGLA ratio, Zn-related gene expression, and gut microbiota alterations (treatment groups are denoted as “biofortified” versus “control”) [20]. Two papers evaluated the same Zn-biofortified wheat as part of a complete diet composed of 75% wheat, with Knez et al. (2018) evaluating the LA:DGLA ratio and Zn-related gene expression, and Reed et al. (2018) evaluating gut microbiota alterations of the same study (treatment groups are denoted as “Low Zn” for the standard wheat control and “High Zn” for the Zn-biofortified wheat) [18,21]. Two papers evaluated chronic dietary Zn deficiency utilizing purified diets, with Reed et al. (2014) evaluating the LA:DGLA ratio and Zn-related gene expression, and Reed et al. (2015) evaluating gut microbiota alterations of the same study (treatment groups are denoted as “Zn adequate” and “Zn deficient”) [13,17]. The characteristics and methods of the studies are described in Table 2.

Table 3 summarizes the main findings of the five selected studies with respect to parameters used for the ZSI, with the main results described in the following subsections.

#### 2.2.1. Zn Consumption

Zn intakes were consistently higher in the Zn-adequate versus Zn-deficient groups in Reed et al. (2014) and Reed et al. (2015) [13,17]. For Knez et al. (2018) and Reed et al. (2018), Zn intakes were consistently lower in the low-Zn group versus the high-Zn group [18,21]. In Beasley et al. (2020), the biofortified group had lower Zn consumption than the control group over the course of the study (21.0 mg compared to 22.1 mg Zn, respectively) [20].

#### 2.2.2. LA:DGLA Ratio

Of the five studies included for the ZSI evaluation, three studies evaluated the erythrocyte LA:DGLA ratio. In Reed et al. (2014), the LA:DGLA was significantly decreased in the Zn-adequate group relative to the Zn-deficient group at weeks 1, 2, and 3, but not significantly different at week 4 [13]. In Knez et al. (2018), there was a significant decrease in the LA:DGLA ratio in subjects on the high Zn wheat-based diet at each timepoint (weeks 2, 4, 6) [21]. In Beasley et al. (2020), in the biofortified group relative to the control group, the LA:DGLA ratio was significantly decreased at two weeks [20].

#### 2.2.3. Zn-Related Gene Expression

Three of the five studies included for ZSI evaluated Zn-related gene expression. In Reed et al. (2014) and Knez et al. (2018), Δ6-desaturase gene expression was significantly altered in the experimental group with increased Zn consumption [13,21]. The gene expression of tested Zn transporters (ZnT1, ZnT5, ZnT7, ZIP4, ZIP6, ZIP9) was significantly downregulated in the high-Zn group compared to the low-Zn group in the Knez et al. (2018) study [21]. For the tested Zn transporters (ZnT1, ZnT5, ZnT7, ZIP6, ZIP9) in Reed et al. (2014), there were no significant changes in gene expression between the Zn-adequate and Zn-deficient groups [13]. There were no significant changes in Zn-related gene expression in Beasley et al. (2020) between the biofortified and control groups [20].

#### 2.2.4. Analysis of the Gut Microbiota

Of the five studies included for ZSI evaluation, three studies evaluated gut (cecal) microbiota modulation. All three studies found changes in β-diversity, whereas two studies (Reed et al. (2015) and Beasley et al. (2020)) found changes in α-diversity between the experimental and control groups [17,18,20]. At the phyla level, Reed et al. (2015) and Beasley et al. (2020) found increased *Firmicutes* and *Proteobacteria* relative abundance between the control and experimental groups [17,20]. In Reed et al. (2018), no significant changes were found at the phyla level between the high-Zn and low-Zn groups [18]. The studies that focused on biofortified wheat found changes in bacterial abundance at the genera level in *Dorea* spp. and *Ruminococcus* spp. between the biofortified and control groups [18,20]. In all three studies, Zn biofortification and/or Zn adequacy was found to be associated with increased SCFA production. Finally, metagenomic potential of the gut microbiota was found to be significantly altered in all three studies [17,18,20].

#### 2.2.5. Additional Biomarkers of Zn Physiological Status

Evaluation of serum, feather, and nail Zn content was done in three of the five studies, and evaluation of liver Zn content was done in one of the five studies. In Reed et al. (2014) and Knez et al. (2018), significant differences were found between serum, feather, and nail Zn content [13,21]. In Beasley et al. (2020), there were no significant changes in serum, feather, nail, or liver Zn content when comparing the biofortified group to the control group [20].

### 2.3. Discussion

The ZSI consists of the combination of the LA:DGLA ratio, expression of Zn-related proteins, and alterations in gut microbiome with respect to dietary Zn intake. In recent years, several in vivo and clinical studies have been conducted to evaluate the efficacy of the LA:DGLA ratio in assessing Zn physiological status with respect to dietary Zn intake [13,21,33,34,35,38]. Given the complexity of Zn metabolism, the association between mRNA gene expression of Zn-related proteins and Zn physiological status can be assessed. Additionally, as Zn is essential for bacteria, the abundance of Zn-dependent microorganisms may be altered in an environment depending on Zn bioavailability [39].

#### 2.3.1. The LA:DGLA Ratio as a Potential Reactive Biomarker of Zn Physiological Status

The previously unexplored biomarker of Zn physiological status related to erythrocyte ∆6-desaturation, the LA:DGLA ratio, was first evaluated in 2014 by Reed et al. [13]. The authors utilized an in vivo model (*Gallus gallus*) sensitive to dietary Zn manipulations [22,40] and found a significant negative correlation between dietary Zn intake and the erythrocyte LA:DGLA ratio. In this original study, subjects were fed either a Zn-adequate control diet (42.3 μg Zn/g) or a Zn-deficient diet (2.5 μg Zn/g) over the course of four weeks [13]. The study found that the cumulative LA:DGLA ratio was noticeably elevated in the Zn-deficient group compared to the Zn-adequate group, indicating the erythrocyte LA:DGLA ratio accurately differentiated Zn status between Zn-adequate and Zn-deficient subjects [13]. Further, differences in the LA:DGLA ratio were noticeable within one week, demonstrating the sensitivity of this biomarker to dietary Zn status and the possibility of using this biomarker for detecting early changes in Zn physiological status that may usually, due to the lack of obvious signs and symptoms, pass unrecognized [13].

This proposed biomarker of Zn physiological status was further evaluated in in vivo studies that studied the effects of Zn-biofortified and nicotianamine-enhanced Zn- and Fe-biofortified wheat on Zn status [20,21]. The animal subjects in these studies consumed a wheat-based diet, which is a representative diet of target Zn-deficient populations. In Knez et al. (2018), subjects were fed a low-Zn diet (standard wheat, 32.8 ± 0.17 μg Zn/g) or high-Zn diet (Zn-biofortified wheat, 46.5 ± 0.99 μg Zn/g) over the course of six weeks [21]. The LA:DGLA ratio was higher in the low-Zn group at all time points measured (weeks 2, 4, and 6), and the difference in Zn dosing in Knez et al. (2018) was only 14 μg Zn/g versus 40 μg Zn/g in Reed et al. (2014) [13,21]. This demonstrated that with only a 14 μg Zn/g differential in dietary Zn content, the LA:DGLA ratio differentiated clearly between treatment groups, thus demonstrating the sensitivity of the biomarker to change in accordance with dietary Zn intake [21]. In Beasley et al. (2020) [20], subjects were given a biofortified diet (nicotianamine-enhanced Zn- and Fe-biofortified wheat) or control (standard wheat) diet, wherein the biofortified subjects had lower Zn consumption than the control subjects over the course of the six-week study (21.0 mg compared to 22.1 mg Zn, respectively). It was found that the LA:DGLA ratio was significantly decreased at week 2 and there was a trend of decreased LA:DGLA from week 4 onwards in the biofortified group relative to the control group [20]. Given the small differences in dietary Zn concentration (<3 μg Zn/g), and that the biofortified group had lower Zn consumption than the control group, the authors posited that the biofortified chickens may have had improved Zn bioavailability due to consumption of increased dietary nicotianamine, although whether nicotianamine or its metabolite (2′-deoxymugineic acid) increase Zn bioavailability requires further investigation [20].

Traditional biomarkers of Zn status, such as Zn in serum and tissues (feather and nail) were also assessed in the aforementioned in vivo studies. Given the wide differences in Zn dietary content in Reed et al. (2014) and Knez et al. (2018), the concentration of Zn in serum, feather, and nail was greater in the treatment groups with higher Zn dietary intake than in the treatment groups with lower Zn intake (*p* < 0.05) [13,21]. In Beasley et al. (2020), Zn concentration in serum, nail, and feather samples were unchanged in the biofortified subjects relative to the control subjects, suggesting that Zn status was unchanged [20]. However, given the small differences in dietary Zn consumption (21.0 mg for biofortified subjects compared to 22.1 mg Zn for control subjects), the traditional biomarkers of Zn status may not have been sensitive enough when compared to the LA:DGLA ratio, where a significant difference in LA:DGLA ratio was found between treatment groups at the two-week timepoint, suggesting differences in Zn status [20]. These observations are in agreement with previous research that suggested the problematic sensitivity of plasma Zn as a biomarker of Zn status, and further highlights the need to develop sensitive biomarkers of Zn status [12,41].

This proposed biomarker of Zn physiological status has been further evaluated in clinical studies and found to change in accordance with dietary Zn intake [33,34,35]. Knez et al. (2017) found that in healthy human adult volunteers, changes in plasma LA:DGLA ratio corresponded to dietary Zn intake [35]. Further, the study found that although plasma Zn concentrations remained unchanged, the LA:DGLA ratio was increased in participants with lower dietary Zn intakes [35]. In 2019, Knez et al. found that subjects with dyslipidemia had inadequate dietary intakes of Zn and a low plasma Zn status. The study also found no correlations between plasma Zn and dietary Zn intake, but found an inverse correlation between dietary Zn intake and the LA:DGLA ratio, reconfirming the sensitivity of the LA:DGLA ratio in humans [38]. The LA:DGLA ratio was assessed in a randomized controlled trial in Beninese children, where a negative association was found between the LA:DGLA ratio and plasma Zn concentration at the study baseline, further supporting the value of the LA:DGLA ratio as a potential biomarker of Zn physiological status [34]. Monteiro et al. (2021) evaluated the association between Zn and polyunsaturated fatty acid (PUFA) intake related to the LA:DGLA ratio, and found an inverse correlation between the LA:DGLA ratio and serum Zn, and associated the LA:DGLA ratio with dietary patterns related to Zn and PUFA intake [33]. Further, King (2018) discussed how in humans, enzymes such as ∆6-desaturase (FADS2, or fatty acid desaturase 2) involved in metabolizing linoleic acid are sensitive to modest changes in dietary Zn [41]. Given that the LA-to-DGLA conversion pathway takes place in the red blood membrane, and red blood cell fatty acid composition is more stable over time within a person and is unaffected by fasting status, future clinical studies should focus on determining the LA:DGLA ratio in the red blood cell fraction instead of the plasma or serum fraction [42,43].

#### 2.3.2. Zn-Related Gene Expression in Relation to Zn Dietary Intake In Vivo

Previous in vivo studies have documented that even mild Zn deficiency can alter Zn transporter gene expression and brush border membrane enzyme activity [14,15]. As Zn exists as a charged, hydrophobic ion, specialized protein transporters are required to move Zn across the plasma membranes for cellular uptake and release. Two Zn transporter families work together to regulate Zn homeostasis in the cell, where ZnT proteins (Zn efflux transporters, SLC30 family) export Zn from the cytoplasm, whereas ZIP proteins (Zn influx transporters, SLC39 family) import Zn into the cytoplasm [44,45,46,47]. ZnT1 is the major Zn export protein, and ZIP4 is the most important Zn import protein, where ZnT1 and ZIP4 expression changes have been associated with the molecular basis of systemic Zn homeostatic regulation [6,48,49]. However, both increased and decreased ZnT and ZIP expression have been demonstrated in response to Zn deficiency [6,45,47].

Gene expression of duodenal Zn-related transporters was assessed in relation to Zn dietary intake. In Reed et al. (2014), there were no significant changes in gene expression of Zn transport proteins between the Zn-adequate and Zn-deficient groups, suggesting that these mRNA gene expression biomarkers may not be sensitive enough to reveal differences in Zn status in a four-week feeding trial, and lack of gene expression changes may have been a compensatory mechanism by the subjects to a Zn-deficient diet [13]. In Knez et al. (2018), which was a six-week feeding trial, the gene expression of tested Zn transporters (ZnT1, ZnT5, ZnT7, ZIP4, ZIP6, ZIP9) were significantly downregulated in the high-Zn group (Zn-biofortified wheat) compared to the low-Zn (control) group [21]. Given that the Knez et al. (2018) study was over a longer period of time, and the differential in Zn concentration between the experimental and control group was not as wide as that in Reed et al. (2014) (46.5 μg/g versus 32.8 μg/g in high-Zn versus low-Zn in Knez et al. (2018), compared to 42 μg/g versus 2.5 μg/g in Zn-adequate versus Zn-deficient), it is possible that the significant changes in Zn transporter gene expression were associated with the longer duration of the feeding trial [13,21].

Hepatic Δ6-desaturase mRNA gene expression was also assessed. Zn is an essential cofactor for the Δ6-desaturase enzyme; thus, Zn deficiency affects the function and gene expression of Δ6-desaturase [13,32,50,51]. In Reed et al. (2014) and Knez et al. (2018), Δ6-desaturase gene expression was significantly altered in the experimental group with higher Zn consumption [13,21]. In Beasley et al. (2020), there were no changes in Δ6-desaturase gene expression, potentially due to the small differences in dietary Zn consumption between the experimental and control groups [20]. Taken together, these findings suggest differential dietary Zn can alter Zn-related gene expression.

#### 2.3.3. Gut Microbiome as a Potential Indicator of Zn Status

Bacteria that colonize the gastrointestinal tract are dependent on minerals such as Zn, where bacterial metabolites can contribute to mineral solubility [19,39]. Additionally, as Zn is essential for bacteria, the abundance of Zn-dependent microorganisms may be dependent on Zn bioavailability [39]. The three studies presented performed 16S rRNA gene sequencing to elucidate the effects of Zn consumption in relation to bacterial phylogeny and taxonomy [17,18,20]. One study, which had the largest differential in Zn content between experimental groups, found a significant decrease in α–diversity with a Zn-deficient diet compared to a Zn-adequate diet (Chao1 for species richness and total observed OTUs for diversity), suggesting that a Zn-depleted environment may lead to a less diverse microbial community, preferentially composed of species that are viable under low Zn conditions [17]. Changes in β-diversity were found in all three studies between the treatment and the control groups, though the change is not necessarily indicative of either beneficial or negative variations in bacterial taxa [17,18,20].

Reed et al. (2015) found an increase in prevalence of *Ruminococcus lactaris*, *Enterococcus* sp., *Clostridium lactatifermentans*, and *Clostridium clostridioforme*, and a decrease in prevalence of *Clostridium indolis* and an unclassified member of the *Bacteroidales* (Unclassified S24–7) in the group that received the Zn-adequate diet compared to the group that received a Zn-deficient diet. With chronic Zn deficiency, a decrease in the prevalence of members of the Firmicutes phylum, such as the genera *Clostridium*, which contains SCFA producers, was also found [17]. Increased SCFA production lowers intestinal luminal pH, which has been associated with preventing proliferation of potentially pathogenic bacteria and increasing Zn bioavailability and uptake [52,53]. Reed et al. (2018) observed an expansion in *L. reuteri*, and members of *Dorea*, *Clostridiales*, unclassified *Clostridiales*, *Ruminococcus*, *Lachnospiraceae*, and unclassified *Lachnospiraceae* genera in the group that received the high-Zn diet compared to the group receiving the low-Zn diet [18]. The *Ruminococcus* and *Clostridiales* genera include species of bacteria that are known SCFA producers [54,55]. Additionally, bacteria from the *Lachnospiraceae* family, such as the *Blautia* genera, are among the main producers of SCFAs [56]. Decreased abundance of *Lachnospiraceae* has been associated with negative health implications resulting from the loss of numerous beneficial functions, such as SCFA production, performed by members of this family [57,58]. LEfSe was utilized in Reed et al. (2018) to investigate significant bacterial biomarkers that could identify differences in the gut microbiota of treatment groups, where a four-fold increase in the *Lactobacillaceae* phyla was found [18]. Members of the *Lactobacillaceae* phyla have been shown to improve gut health by producing SCFAs; decreasing the colonization of pathogenic microorganisms, such as *Salmonella* spp. and enteropathogenic *E. coli*; and increasing villus surface area and goblet cell number per villi [59,60,61]. In Beasley et al. (2020), the authors observed an increase in the abundance of the *Actinobacteria* phylum and a reduction in the abundance of *Firmicutes* and *Proteobacteria* in the biofortified group compared to the control group [20]. An increase in abundance of *Actinobacteria* phyla has been associated with the consumption of plant dietary fiber, suggesting a potential beneficial effect of this phylum on intestinal health [19]. All studies suggested alterations in gut microbiota composition in association with the abundance and capacity of resident intestinal microbiota to provide SCFAs in the lower Zn intake or lower Zn bioavailability group, which can further deplete Zn bioavailability in an already Zn-insufficient state.

Through metagenomic analysis, the presented three studies found alterations in predicted Kyoto Encyclopedia of Genes and Genomes (KEGG) pathways. Reed et al. (2014) found decreased expression of pathways related to mineral (i.e., Zn) absorption and carbohydrate digestion and fermentation, where the latter pathway may also contribute to the depression in SCFA production, which has been associated with improving Zn bioavailability [17]. KEGG pathway analysis in Reed et al. (2018) found six bacterial biosynthetic pathways to be depleted in the low-Zn diet group, where pathways responsible for bile acid production, cytochrome p450 activity, and glycan metabolism were found to be significantly depleted and posited to reflect the decreased concentration of bioavailable Zn in the intestinal lumen [18]. Beasley et al. (2020) found the metagenomic potential of microbial glycolysis/gluconeogenesis significantly increased and microbial tropane piperidine and pyridine alkaloid biosynthesis significantly decreased in the biofortified group’s microbial populations relative to the control [20]. Altogether, the microbial effects presented in these studies suggest that a significant remodeling of the intestinal microbiota occurs in animal subjects receiving diets with differential content and bioavailability of Zn.

## 3. Development of the ZSI

### 3.1. Statistical Strategy for Creating the Zinc Status Index (ZSI)

Using data from dietary-controlled experiments in three studies conducted in *Gallus gallus*, data were obtained for three types of predictors: the LA:DGLA ratio, gene expression of selected genes associated with Zn metabolism, and microbiome factors. The training sample utilized data from Knez et al. (2017), Reed et al. (2018), and Beasley et al. (2020). The studies selected investigated differential dietary Zn in the context of a complete diet. A training sample of n = 59 and a total of 25 potential predictors for the Zn status, including LA:DGLA, eight Zn-dependent genes, and 16 bacteria genera, was utilized for ZSI prototype development (Table 4). Note that not all 59 samples have data for all 25 predictors.

We used these 25 variables as predictors for diet status (control vs. Zn biofortified) and fitted a binary classifier. Specifically, we used the logistic regression and the CART (classification and regression tree) methods. We eliminated from the fitted model any variables that had no predictive power (namely, they were not associated with the binary diet variable, meaning changes in dietary Zn intake). The remaining predictors were used to define our ZSI, which allowed us to predict the probability of Zn-adequacy or -deficiency status. Additionally, when obtaining the prediction formulas for this study and fitting the logistic model, we added the experiment as a factor to control for differences between experiments. Supposing that we have a total of four predictors (x_1_, …, x_4_) that were found to be significantly associated with the diet variable, then the logistic formula is

(1) $$\log\frac{p}{1-p}{=\beta }_{0}{+\beta }_{1}x_{1}{+\beta }_{2}x_{2}{+\beta }_{3}x_{3}{+\beta }_{4}x_{4}$$

where *p* is the probability that a subject has an adequate level of Zn, and β_i_ is the coefficient obtained from our training data. Then, the index was simply obtained by measuring only x_1_, …, x_4_ for new samples, entering them into the formula, and determining Zn physiological status based on whether log(*p*/1 − *p*) was greater than a certain threshold. For example, we could set the Zn status as deficient if log_2_(*p*/1 − *p*) < −2, that is, the odds that the Zn level is adequate, was less than ¼ and the probability that the level of Zn was adequate was 0.25. Stricter thresholds could be used.

Our index is probabilistic in nature, so given the data for the selected predictors (or some of them), we could determine the probability of whether the Zn levels were adequate or deficient.

### 3.2. Examples of the ZSI as a Predictor of Zn Status

We obtained the following estimations (examples) for the probability that a hypothetical human or animal subject is Zn adequate. In the following examples, *p* ranged from 0 to 1, and we set preliminary quintiles for estimated Zn status as shown in Table 5.

**Example** **1.** *(Relevant for humans and animal models): Using data from our previous experiments, we obtained the following estimation for the probability that a subject is Zn deficient:*

(2) $$\log\frac{p}{1-p}=5.18\;-\;0{.015x}_{1}-\;0{.26x}_{2}\;+43{.39x}_{3}\;$$

*where x_1_ is the LA:DGLA level, x_2_ is the Δ6-desaturase expression, x_3_ is the Blautia relative abundance, and p is the probability that a subject has an adequate level of Zn.*

For example 1, as depicted in Table 6, hypothetical subject A, whose LA:DGLA ratio is at the 50th percentile (x_1_ = 50) and whose Δ6-desaturase expression levels and *Blautia* relative abundance are equal to the median (x_2_ = 192, x_3_ = 0.021), the predicted probability that subject A has an adequate Zn level is 0.59, with an estimated Zn status of mildly Zn deficient. If subject B has an LA:DGLA level equal to the 20th percentile (x_1_ = 38) and the Δ6-desaturase and *Blautia* relative abundance are equal to the median, then the probability that subject B is Zn adequate is 0.64, corresponding to an estimated minimally Zn-adequate status. For subject C, the LA:DGLA level and *Blautia* relative abundance are the same as subject A, but subject C has a Δ6-desaturase expression in the 80th percentile (x_2_ = 249), so the predicted probability that subject C is Zn adequate is 0.25, with an estimated moderately Zn-deficient status. Finally, if the LA:DGLA level and Δ6-desaturase expression remain the same as subject A, but subject D’s *Blautia* relative abundance is at the 80th percentile (x_3_ = 0.035), the probability that subject D is Zn adequate increases to 0.73, with an estimated minimally Zn-adequate status.

**Example** **2.** *(Relevant for humans and animal models): Using data from our previous experiments, we obtained the following estimation for the probability that a subject is Zn deficient:*

(3) $$\log\frac{p}{1-p}=3.95\;-\;0{.016x}_{1}-\;0{.31x}_{2}\;+145{.7x}_{3}\;$$

*where x_1_ is the LA:DGLA level, x_2_ is the Δ6-desaturase expression, x_3_ is the unclassified Lachnospiraceae relative abundance, and p is the probability that a subject has adequate level of Zn.*

For example 2, as depicted in Table 7, hypothetical subject A, whose LA:DGLA is at the 50th percentile (x_1_ = 50) and whose Δ6-desaturase expression levels and unclassified *Lachnospiraceae* relative abundance are equal to the median and 20th percentile (x_2_ = 192 and x_3_ = 0.013, respectively), the predicted probability that subject A has an adequate Zn level is 0.33, with an estimated Zn status of moderately Zn deficient. If subject B has a LA:DGLA level equal to the 20th percentile (x_1_ = 38) and the Δ6-desaturase and unclassified *Lachnospiraceae* relative abundance are equal to that of subject A, then the probability that subject B is Zn adequate is 0.37, corresponding to an estimated moderately Zn-adequate status. For subject C, the LA:DGLA level and unclassified *Lachnospiraceae* relative abundance are the same as subject A, but subject C has a Δ6-desaturase expression that is in the 20th percentile (x_2_ = 153), where the predicted probability that subject C is Zn adequate is 0.62, with an estimated minimally Zn-deficient status. Finally, if the LA:DGLA level and Δ6-desaturase expression remain the same as subject A, but subject D’s unclassified *Lachnospiraceae* relative abundance is at the 80th percentile (x_3_ = 0.034), the probability that subject D is Zn adequate increases to 0.90, with an estimated Zn-adequate status.

**Example** **3.** *(Relevant for animal models): Using data from our previous experiments, we obtained the following estimation for the probability that an animal subject is Zn-deficient:*

(4) $$\log\frac{p}{1-p}=\;15.9\;-0{.05x}_{1}\;-0{.03x}_{2}\;-\;0{.26x}_{3}\;$$

*where x_1_ is the LA:DGLA level, x_2_ is the Δ6-desaturase expression, x_3_ is the ZIP9 expression, and p is the probability that a subject has adequate level of Zn.*

For example 3, as depicted in Table 8, where LA:DGLA is at the 80th percentile (x_1_ = 70) and the Δ6-desaturase and ZIP9 expression levels are equal to the median (x_2_ = 197, x_3_ = 31), the predicted probability that subject A has an adequate Zn level is 0.28, with an estimated moderately Zn-deficient status. If animal subject B has a LA:DGLA level at the 20th percentile (x_1_ = 38) and the Δ6-desaturase and ZIP9 expression remain the same compared to animal subject A, then the probability that subject B is Zn adequate is 0.67, corresponding to an estimated minimally Zn-adequate status. For animal subject C, the LA:DGLA level and ZIP9 are the same as animal subject A, but animal subject C has a Δ6-desaturase expression that is in the 20th percentile (x_2_ = 153), so the predicted probability that subject C is Zn adequate is 0.6, with an estimated mildly Zn-deficient status. Finally, if the LA:DGLA level and Δ6-desaturase expression remain the same as animal subject A, but subject D’s ZIP9 expression level is at the 90th percentile (x_3_ = 45), the probability that animal subject D is Zn adequate drops to 0.016, with an estimated severely Zn-deficient status.

### 3.3. Zinc Status Index as an Accurate Predictor of Zn Physiological Status

**Figure 1:**
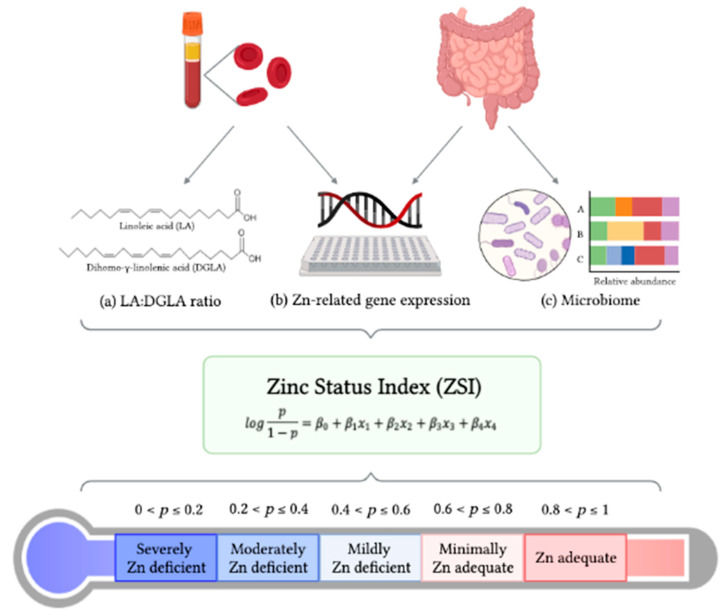
Schematic of ZSI prediction model development. Three pillars, (**a**) LA:DGLA ratio, (**b**) Zn-related gene expression, and (**c**) gut microbiome profile, were utilized for development of the ZSI. Based on our initial ZSI prediction model, we may set preliminary quintiles for Zn status levels based on the predicted probability of Zn adequacy.

Zn is an essential mineral with catalytic, structural, and regulatory functions with sophisticated homeostatic control, making it difficult to identify Zn inadequacy [12,62]. It remains a scientific challenge to obtain an accurate picture of Zn status of both various population groups and individuals [63]. Considering the complexity of Zn metabolism, the establishment of a panel of biochemical indices is necessary to reliably assess Zn status, especially in cases of mild to moderate Zn deficiency. As such, we developed the ZSI prediction model, which consists of a three-pillar formula: (1) the LA:DGLA ratio, (2) mRNA gene expression of Zn-related proteins, and (3) fecal microbial ecology profiling. The formula provides a clear and accurate measurement of Zn physiological status. Each of the three pillars has been shown to be altered with changes in dietary Zn intake and Zn bioavailability [13,17,18,20,21,35]. To illustrate the potential contribution of biomarkers other than the LA:DGLA ratio, consider hypothetical subject 2D, for example. In Table 7, we see that the predicted probability of Zn adequacy for this subject is 0.9 (estimated Zn adequate status). Removing the *Lachnospiraceae* predictor from Equation (3) yields a predicted value of 0.06 (corresponding to an estimated severely Zn-deficient status). It is clear that a model with gene expression and microbiome biomarkers in addition to the LA:DGLA ratio can have a substantial impact on the accuracy of the ZSI. Our ZSI will improve the understanding of Zn nutrition, physiological status, and severity of potential deficiency (Figure 1).

Three examples utilizing our prototype ZSI to estimate Zn status were provided. Examples 1 and 2 can be utilized for both clinical trials (human studies) and animal studies, as LA:DGLA level and Δ6-desaturase (FADS2) gene expression can be obtained from blood samples (erythrocyte fraction) and gut microbiome profiling can be obtained from fecal samples. Example 3 is most relevant for animal models due to the invasive nature of sample collection (duodenal sample) for ZIP9 gene expression. When comparing subjects A and B from our three examples, where only the LA:DGLA level (x_1_) is changed versus the other predictors (x_2_ and x_3_), the decrease in LA:DGLA level was associated with an improvement in Zn status, in line with previous experiments due to the Zn requirement of Δ6-desaturase in the LA-to-DGLA conversion pathway [13,21,64]. When comparing subject A to subject C in all three examples, where the Δ6-desaturase expression level (x_2_) is altered and the other predictors remain the same (x_1_ and x_3_, respectively), lower Δ6-desaturase expression was found to be associated with improved Zn status, supported by previous studies where Δ6-desaturase gene expression was significantly altered with differential dietary Zn consumption [21]. In example 3, when comparing subject A to subject D, where ZIP9 expression (x_3_) is higher in subject D and LA:DGLA level and Δ6-desaturase (x_1_ and x_2_, respectively) remain the same, the estimated Zn status was severely Zn deficient (*p* = 0.016) in subject D versus moderately Zn deficient (*p* = 0.28) for subject A. This aligns with previous studies where Zn transporter gene expression was significantly altered with differential Zn status [21]. In example 1, if the LA:DGLA level (x_1_) and Δ6-desaturase expression (x_2_) remain the same as subject A, but subject D’s *Blautia* relative abundance is at the 80th percentile (x_3_ = 0.035) versus the 50th percentile (x_3_ = 0.021) for subject A, subject D’s estimated Zn status was minimally Zn adequate (*p* = 0.73) versus subject A’s estimated Zn status of mildly Zn deficient (*p* = 0.59). The *Blautia* genera includes SCFA producers, where SCFA activity has been associated with reducing levels of inflammatory markers and interacting with the host immune system [56]. Further, when comparing subjects A and D in example 3, where only the unclassified *Lachnospiraceae* abundance (x_3_) is changed and LA:DGLA level and Δ6-desaturase gene expression (x_1_ and x_2_, respectively) remain the same, the increase in unclassified *Lachnospiraceae* abundance from the 20th percentile in subject A to the 80th percentile in subject D was associated with an improvement in predicted Zn status from moderately Zn deficient to Zn adequate. Both *Blautia* and unclassified *Lachnospiraceae* belong to the *Lachnospriaceae* family, and *Lachnospiraceae* play a key role in carbohydrate metabolism [58,65]. Metagenomic analysis found that a Zn-deficient state was associated with decreased expression of pathways related to carbohydrate digestion and fermentation, which may contribute to a decrease in SCFA production [17]. Given that bacterial abundance of the *Lachnospiraceae* family was altered based on level of Zn adequacy and the ZSI found bacteria of this family to have significant predictive power, future studies should target *Lachnospiraceae* for further refinement of the ZSI. Future clinical trials should take into account the combination of the LA:DGLA level in red blood cells, Δ6-desaturase gene expression in red blood cells, and fecal microbial abundance of bacteria in the *Lachnospiraceae* family for accurate assessment of Zn physiological status.

Our three-pillar ZSI concept demonstrated that the LA:DGLA ratio, Zn-related gene expression, and microbiome analyses were predictive factors of Zn status; however, our prediction formula was obtained using data from in vivo studies and a relatively small sample size (n = 59). It is possible these parameters may be part of a larger set of predictive parameters, and future research may further elucidate which parameters have the highest predictive power. Given that each experiment will yield different LA:DGLA ratios, gene expression levels, and microbiome profile, a calibration tool will need to be built into the model. For example, for subject A in example 3, shown in Table 6, the 80th percentile of LA:DGLA ratio is approximately 70, but if another subject from another study has an LA:DGLA of 50, we need to know what this value would translate to if the subject were analyzed with a different experimental batch. Microbiome profiles may differ between target populations [65,66], where there may be differences in bacterial abundance associated with differential Zn status between specific target populations. Additionally, there is future potential for minimizing the cost of each test. For example, if the observed LA:DGLA ratio is sufficiently high (or low) based on our predictive formula to conclude the Zn adequacy level with high probability, then further gene expression or microbiome analyses may not be necessary. As shown in example 3, the combination of the LA:DGLA level and Δ6-desaturase and ZIP9 expression had sufficient predictive power where fecal microbial profiling was not included. This demonstrates the versatility and flexibility of the ZSI concept. After further refinement of the ZSI prediction model utilizing data from future studies, a baseline for calibration can be built to standardize for potential batch effects associated with differences in experimental dates, sites, and/or target populations.

Altogether, the ZSI was shown to be a highly sensitive Zn status predictor, predictive of various degrees of Zn adequacy (or inadequacy). The current ZSI is a prototype and will evolve as more data emerge. Further, our eventual goal is to use the ZSI to predict Zn physiological status at both the individual and population levels. Utilization of the ZSI will ultimately lead to more precise assessment of the effects of dietary Zn interventions and medical outcomes.

## 4. Conclusions

We present the ZSI prototype as a strategy to better understand Zn nutrition in the context of a complete diet. Our evidence demonstrates the potential of the ZSI as an accurate predictor of Zn physiological status that is responsive to dietary Zn changes. The ZSI can be used to assess the efficacy of dietary interventions in target populations, for example, in the context of the assessment of Zn-biofortified staple food crops, relevant dietary supplements or fortifiers, and other nutritional approaches that are used to improve Zn status. Zn deficiency is often missed due to the inflammation status of the subject (and when serum/plasma Zn concentrations are used as Zn physiological status), which is especially pertinent in vulnerable populations. Thus, the development and usage of the ZSI is highly relevant for the accurate measurement of Zn physiological status. Further studies are warranted to further train and refine the ZSI model.

## Figures and Tables

**Table 1:** The DNA sequences of primers used in this study: ZnT1, zinc transporter 1; ZnT5, zinc transporter 5; ZnT7, zinc transporter 7; ZIP1, zinc transport protein 1; ZIP4, zinc transport protein 4; ZIP6, zinc transport protein 6; ZIP9, zinc transport protein 9.

Analyte	Organ	Forward Primer (5′–3′)	Reverse Primer (5′–3′)	Base Pair	GI Identifier
ZnT1	duodenum	GGTAACAGAGCTGCCTTAACT	GGTAACAGAGCTGCCTTAACT	105	54109718
ZnT5	duodenum	TGGTTGGTATCTGTGCCTTTAG	GGCTGTGTCCATGGTAAGATT	99	56555150
ZnT7	duodenum	GGAAGATGTCAGGATGGTTCA	CGAAGGACAAATTGAGGCAAAG	87	56555152
ZIP1	duodenum	TGCCTCAGTTTCCCTCAC	GGCTCTTAAGGGCACTTCT	144	XM_015298606.1
ZIP4	duodenum	TCTCCTTAGCAGACAATTGAG	GTGACAAACAAGTAGGCGAAAC	95	107050877
ZIP6	duodenum	GCTACTGGGTAATGGTGAAGAA	GCTGTGCCAGAACTGTAGAA	99	66735072
ZIP9	duodenum	CTAAGCAAGAGCAGCAAAGAAG	CATGAACTGTGGCAACGTAAAG	100	237874618
Δ6-desaturase	liver	GGCGAAAGTCAGCCTATTGA	AGGTGGGAAGATGAGGAAGA	93	261865208
18S	duodenum, liver	GCAAGACGAACTAAAGCGAAAG	TCGGAACTACGACGGTATCT	100	7262899

**Table 2:** Characteristics and methods of studies assessed for the ZSI: LA:DGLA, Zn-related gene expression, and Zn status effects on gut microbiota modulation.

Reference	Animal Model	Number of Subjects	Treatment or Intervention	Duration (Weeks)	Zn Status Measures			
					LA:DGLA (Erythrocyte)	Zn-Related Gene Expression	Gut Microbiota Evaluation Method	Other (Not Included in ZSI)
Beasley et al., 2020 [20]	Cornish Cross broiler (*Gallus gallus*)	30 (n = 15 per group)	Nicotianamine-enhanced Zn- and Fe-biofortified wheat (*Triticum aestivum* L*.)*	6	Yes	ZnT1 ZnT5 ZnT7 ZIP1 ZIP4 ZIP6 ZIP9 Δ6-desaturase	16s rRNA gene sequencing	Serum Liver Nail Feathers
Knez et al., 2018 [21]	Cornish Cross broiler (*Gallus gallus*)	30 (n = 15 per group)	Zn-biofortified wheat (*Triticum aestivum*)	6	Yes	ZnT1 ZnT5 ZnT7 ZIP4 ZIP6 ZIP9 Δ6-desaturase	N/A	Serum Nail Feathers
Reed et al., 2018 [18]	Cornish Cross broiler (*Gallus gallus*)	30 (n = 15 per group)	Zn-biofortified wheat (*Triticum aestivum*)	6	Yes	ZnT1 ZnT5 ZnT7 ZIP4 ZIP6 ZIP9 Δ6-desaturase	16s rRNA gene sequencing	Serum Nail Feathers
Reed et al., 2015 [17]	Cornish Cross broiler (*Gallus gallus*)	24 (n = 12 per group)	Zn-adequate control diet versus Zn-deficient diet (Zn carbonate as Zn source)	4	Yes	ZnT1 ZnT5 ZnT7 ZIP6 ZIP9 Δ6-desaturase	16s rRNA gene sequencing	Serum Nail Feathers
Reed et al., 2014 [13]	Cornish Cross broiler (*Gallus gallus*)	24 (n = 12 per group)	Zn-adequate control diet versus Zn-deficient diet (Zn carbonate as Zn source)	4	Yes	ZnT1 ZnT5 ZnT7 ZIP6 ZIP9 Δ6-desaturase	N/A	Serum Nail Feathers

**Table 3:** Results of studies assessed for the ZSI: LA:DGLA, Zn-related gene expression, and Zn status effects on gut microbiota modulation.

Reference	Zn Content (μg Zn/g)	Zn Status Measures			
		LA:DGLA (Erythrocyte)	Zn-Related Gene Expression	Gut Microbiota Modulation	Other (Not Included in ZSI)
Beasley et al., 2020 [20]	Control: 16.6 ± 0.06 (standard wheat) Biofortified: 19.2 ± 0.03 (nicotianamine-enhanced Zn- and Fe-biofortified wheat)	In the biofortified relative to the control group: ↓ LA:DGLA at 2 weeks ↓ LA:DGLA at 4 weeks onwards (trend, not significant)	In the biofortified relative to the control group: ↔ ZnT1 ↔ ZnT5 ↔ ZnT7 ↔ ZIP1 ↔ ZIP4 ↔ ZIP6 ↔ ZIP9 ↔ Δ6-desaturase	In the biofortified relative to the control group: ↓ α-diversity Change in β-diversity At the phyla level: ↑ 1.9-fold the proportion of *Actinobacteria* ↓ 1.2- and 2.0-fold, respectively, the proportion of *Firmicutes* and *Proteobacteria* At the family level: ↑ abundance of *Enterococcaceae* ↓ 1.7-fold the proportion of *Lachnospiraceae* At the genera level: ↑ *Enterococcus* abundance ↓ *Dorea* abundance ↑ 1.9- and 1.5-fold, respectively, proportion of *Bifidobacterium* and *Lactobacillus* ↓ proportion of *Streptococcus* (1.7-fold), *Coprococcus* (1.4-fold), *Ruminococcus* (1.2-fold) *Faecalibacterium* (2-fold), and *Escherichia* (2-fold)	In the biofortified relative to the control group: ↔ Serum ↔ Liver ↔ Nail ↔ Feathers
Knez et al., 2018 [21]	Low Zn: 32.8 ± 0.17 (standard wheat) High Zn: 46.5 ± 0.99 (Zn-biofortified wheat)	In the high-Zn relative to the low-Zn group: ↓ LA:DGLA (2 weeks onwards)	In the high-Zn relative to the low-Zn group: ↓ ZnT1 ↓ ZnT5 ↓ ZnT7 ↓ ZIP4 ↓ ZIP6 ↓ ZIP9 ↓ Δ6-desaturase	See Reed et al., 2018 [18]	In the high-Zn relative to the low-Zn group: ↑ Serum Zn (2 weeks onwards) ↑ Feather Zn ↑ Nail Zn
Reed et al., 2018 [18]	Low Zn: 32.8 ± 0.17 (standard wheat) High Zn: 46.5 ± 0.99 (Zn-biofortified wheat)	See Knez et al., 2018 [21]	See Knez et al., 2018 [21]	In the high-Zn relative to the low-Zn group: ↔ α-diversity Change in β-diversity At the phyla level: ↔ *Firmicutes*, *Actinobacteria*, and *Proteobacteria* At the genera level: ↑ *Dorea*, *Clostridiales*, unclassified Clostridiales, *Ruminococcus, Lachnospiraceae,* and unclassified *Lachnospiraceae* ↓ *Lactococcus, Verrucomicrobium, Bacteroides, Bacteroidales,* and unclassified *Bacteroidales* At the species level: ↑ *Lactobacillus reuteri* ↓ *Akkermansia muciniphila*	See Knez et al., 2018 [21]
Reed et al., 2015 [17]	Zn deficient: 2.5 ± 0.02 Zn adequate (control): 42 ± 0.25	See Reed et al., 2014 [13]	See Reed et al., 2014 [13]	In the Zn-adequate relative to the Zn-deficient group: ↑ α-diversity (species richness and diversity) Changes (expansion) in β-diversity At the phyla level: ↑ *Firmicutes* ↓ *Proteobacteria* At the family level: ↑ *Peptostreptococcaceae* and unclassified *Clostridiales* ↓ *Enterococcaceae* and *Enterobacteriaceae* At the genera level: ↑ unclassified *Clostridiales* and unclassified *Peptostreptococcaceae* ↓ *Enterococcus*, unclassified *Enterococcus*, unclassified *Enterobacteriaceae*, and unclassified *Ruminococcaceae* At the species level: ↑ *Ruminococcus lactaris*, *Enterococcus* sp., *Clostridium lactatifermentans*, and *Clostridium clostridioforme* ↓ *Clostridium indolis* and an unclassified member of the *Bacteroidales* (Unclassified S24–7)	See Reed et al., 2014 [13]
Reed et al., 2014 [13]	Zn deficient: 2.5 ± 0.02 Zn adequate (control): 42 ± 0.25	In the Zn-adequate relative to the Zn-deficient group: ↓ LA:DGLA (1 week onwards)	In the Zn-adequate relative to the Zn-deficient group: ↔ ZnT1 ↔ ZnT5 ↔ ZnT7 ↔ ZIP6 ↔ ZIP9 ↑ Δ6-desaturase	See Reed et al., 2015 [17]	In the Zn-adequate relative to the Zn-deficient group: ↑ Serum Zn ↑ Feather Zn ↑ Nail Zn

↔no change; ↑ increased; ↓ reduced.

**Table 4:** Potential predictors of Zn status used in ZSI development We currently have a training sample of n = 59 *Gallus gallus* and a total of 25 potential predictors for Zn status, including LA:DGLA, eight genes, and 16 bacteria genera.

LA:DGLA	Zn-Related Gene Expression	Gut Bacteria Genera
LA:DGLA ratio in erythrocyte	ZnT1 ZnT5 ZnT7 ZIP1 ZIP4 ZIP6 ZIP9 Δ6-desaturase	*Anaerotruncus* *Bifidobacterium* *Blautia* *Coprococcus* *Escherichia* *Faecalibacterium* *Lactobacillus* *Oscillospira* *Ruminococcus* *Streptococcus* *Sutterella* unclassified *Clostridiales* unclassified *Enterobacteriaceae* unclassified *Lachnospiraceae* unclassified *Ruminococcaceae* Family: *Lachnospiraceae*

**Table 5:** Estimated Zn status based on preliminary ranges of predicted probability (*p*) of Zn adequacy.

Predicted Probability of Zn Adequacy *(p)*	Estimated Zn Status
0 ≤ *p* ≤ 0.2	Severely Zn deficient
0.2 < *p* ≤ 0.4	Moderately Zn deficient
0.4 < *p* ≤ 0.6	Mildly Zn deficient
0.6 < *p* ≤ 0.8	Minimally Zn adequate
0.8 < *p* ≤ 1	Zn adequate

**Table 6:** Predicted probability of Zn adequacy of hypothetical subjects using the above ZSI example 1 ^1^.

Hypothetical Subject	LA:DGLA (x_1_)		Δ6-Desaturase (x_2_)		*Blautia* (x_3_)		Predicted Probability of Zn Adequacy (*p*)	Estimated Zn Status
	Percentile	Value (AU)	Percentile	Value (AU)	Percentile	Value (AU)		
Subject 1A	50	50	50	192	50	0.021	0.59	Mildly Zn deficient
Subject 1B	20	38	50	192	50	0.021	0.64	Minimally Zn adequate
Subject 1C	50	50	80	249	50	0.021	0.25	Moderately Zn deficient
Subject 1D	50	50	50	192	80	0.035	0.73	Minimally Zn adequate

^1^ Note that in all these hypothetical scenarios we assume that the data have been standardized relative to a reference experiment.

**Table 7:** Predicted probability of Zn adequacy of hypothetical subjects using the above ZSI example 2 ^1^.

Hypothetical Subject	LA:DGLA (x_1_)		Δ6-Desaturase (x_2_)		Unclassified *Lachnospiraceae* (x_3_)		Predicted Probability of Zn Adequacy (*p*)	Estimated Zn Status
	Percentile	Value (AU)	Percentile	Value (AU)	Percentile	Value (AU)		
Subject 2A	50	50	50	192	20	0.013	0.33	Moderately Zn deficient
Subject 2B	20	38	50	192	20	0.013	0.37	Moderately Zn deficient
Subject 2C	50	50	20	153	20	0.013	0.62	Minimally Zn adequate
Subject 2D	50	50	50	192	80	0.034	0.90	Zn adequate

^1^ Note that in all these hypothetical scenarios we assume that the data have been standardized relative to a reference experiment.

**Table 8:** Predicted probability of Zn adequacy of hypothetical animal subjects using the above ZSI example 3 ^1^.

Hypothetical Subject	LA:DGLA (x_1_)		Δ6-Desaturase (x_2_)		ZIP9 (x_3_)		Predicted Probability of Zn Adequacy (*p*)	Estimated Zn Status
	Percentile	Value (AU)	Percentile	Value (AU)	Percentile	Value (AU)		
Subject 3A	80	70	50	197	50	31	0.28	Moderately Zn deficient
Subject 3B	20	38	50	197	50	31	0.67	Minimally Zn adequate
Subject 3C	80	70	20	153	50	31	0.60	Mildly Zn deficient
Subject 3D	80	70	50	197	90	45	0.016	Severely Zn deficient

^1^ Note that in all these hypothetical scenarios we assume that the data have been standardized relative to a reference experiment.
